# Global gene expression profiling of *Plasmodium falciparum *in response to the anti-malarial drug pyronaridine

**DOI:** 10.1186/1475-2875-10-242

**Published:** 2011-08-18

**Authors:** Kanyanan Kritsiriwuthinan, Sastra Chaotheing, Philip J Shaw, Chayaphat Wongsombat, Porntip Chavalitshewinkoon-Petmitr, Sumalee Kamchonwongpaisan

**Affiliations:** 1 National Center for Genetic Engineering and Biotechnology (BIOTEC), National Science and Technology Development Agency (NSTDA), Thailand Science Park, Pathumthani 12120, Thailand; 2 Department of Protozoology, Faculty of Tropical Medicine, Mahidol University, Bangkok 10400, Thailand

**Keywords:** Pyronaridine, Chloroquine, microarray, gene expression, *Plasmodium falciparum*

## Abstract

**Background:**

Pyronaridine (PN) and chloroquine (CQ) are structurally related anti-malarial drugs with primarily the same mode of action. However, PN is effective against several multidrug-resistant lines of *Plasmodium falciparum*, including CQ resistant lines, suggestive of important operational differences between the two drugs.

**Methods:**

Synchronized trophozoite stage cultures of *P. falciparum *strain K1 (CQ resistant) were exposed to 50% inhibitory concentrations (IC_50_) of PN and CQ, and parasites were harvested from culture after 4 and 24 hours exposure. Global transcriptional changes effected by drug treatment were investigated using DNA microarrays.

**Results:**

After a 4 h drug exposure, PN induced a greater degree of transcriptional perturbation (61 differentially expressed features) than CQ (10 features). More genes were found to respond to 24 h treatments with both drugs, and 461 features were found to be significantly responsive to one or both drugs across all treatment conditions.

Filtering was employed to remove features unrelated to primary drug action, specifically features representing genes developmentally regulated, secondary stress/death related processes and sexual stage development. The only significant gene ontologies represented among the 46 remaining features after filtering relate to host exported proteins from multi-gene families.

**Conclusions:**

The malaria parasite's molecular responses to PN and CQ treatment are similar in terms of the genes and pathways affected. However, PN appears to exert a more rapid response than CQ. The faster action of PN may explain why PN is more efficacious than CQ, particularly against CQ resistant isolates. In agreement with several other microarray studies of drug action on the parasite, it is not possible, however, to discern mechanism of drug action from the drug-responsive genes.

## Background

Malaria is a parasitic disease accounting for almost one million deaths per year, mostly among children in Sub-Saharan Africa, Asia, Central and South America [1]. During the last two decades, the incidence of malaria has remained unacceptably high. A major factor in the continuing burden of malaria is the spread of parasites resistant to front-line anti-malarials such as chloroquine and sulphadoxine/pyrimethamine [2], and perhaps in the near future, artemisinin [3]. New anti-malarial drugs with different modes of action are needed to overcome drug-resistant parasites.

**Figure 1:**
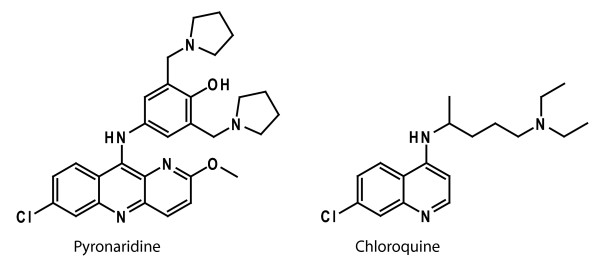
**Chemical structures of Pyronaridine (PN) and Chloroquine (CQ)**.

Pyronaridine (PN) is a Mannich base 9-anilinoacridine structurally related to chloroquine (CQ) (Figure 1). PN was one of the earliest synthetic anti-malarial drugs first developed in China in the late 1970s [4,5]. It is a highly effective blood schizonticidal agent, even against multidrug-resistant lines of *Plasmodium falciparum *[6,7]. In addition to its schizonticidal activity, it has been shown to possess an *in vitro *gametocytocidal effect in *P. falciparum *[8]. It is also effective against *Plasmodium vivax *[9], and *Plasmodium ovale *and *Plasmodium malariae *[10]. Furthermore, it has been demonstrated to be effective in Phase III human trials for treating *P. falciparum *malaria in SE Asia and Africa [11]. Despite extensive reports of its highly potent anti-malarial activity, few studies have been conducted to determine the drug's mechanism of action. Based on the fact that 9-anilinoacridines are potent anti-cancer drugs that target DNA topoisomerase II (TOPO II), it was proposed that PN acted in a similar manner by inhibiting parasite TOPO II [12]. However, it was later shown that parasite TOPO II inhibition by PN is insignificant *in situ *[13]. Auparakkitanon *et al *[14] later demonstrated that the primary mode of action of PN is similar to that of CQ, namely inhibition of α-haematin formation, enhanced haematin-induced red blood cell lysis and interference with glutathione-dependent heme degradation. The hypothesis of shared mode of action is further supported by the electron microscopic examination of PN-treated *P. falciparum *from an infected *Aotus *(owl monkey) [15], which revealed that like CQ, the earliest morphological change induced by PN is the appearance of abnormal vesicles in the food vacuole. Despite the hypothesized similarity of drug action, CQ-resistant field isolates are not cross-resistant to PN, implying that there are some important pharmacological differences between the two drugs [7]. From the available information, it is not known whether the differences between CQ and PN efficacy exist because of differences in mode of action, or differences in cellular resistance mechanisms, e.g., to what extent PN is a substrate for *Pf*CRT-mediated transport compared with CQ.

In this study, a better understanding of the mechanism of action of PN was sought, including the reason why PN is so effective against CQ-resistant parasites, despite the proposed similarity of drug action for PN and CQ. To help answer these questions, DNA microarrays were employed to measure global gene expression changes of a CQ-resistant strain of *P. falciparum *in response to individual 50% inhibitory concentrations of PN and CQ. There is extensive overlap in the genes affected by both drugs, although PN appears to elicit a more rapid response by the parasite than CQ. The majority of genes changing expression in response to both drugs are likely to be the result of developmental arrest and secondary stress/death related cellular processes; hence, there are very few genes which can be considered as specifically PN and CQ drug responsive.

## Methods

### Microarray construction

DNA microarrays were used containing 8,088 70-mer oligonucleotide probes representing the majority of annotated open reading frames (ORF) as listed in PlasmoDB 4.4 [16]. The oligo probes were printed on poly-lysine coated glass slides using a new generation ultra fast, linear servo driven DeRisi microarrayer controlled by an ArrayMaker software [17].

### Parasite culture and drug treatment

K1 (chloroquine-resistant) strain of *P. falciparum *was used for all experiments. This strain was selected since it is representative of the CQ-resistant parasite population where PN is likely to be employed as an anti-malarial in the future. Moreover, PN resistant parasites have not yet been isolated from the field, nor are there any reports of laboratory resistance for *P. falciparum*. Parasites were cultured *in vitro *as described previously in [18]. Culture synchronization was obtained by lysing mature parasites with 5% sorbitol for two consecutive cell cycles [19]. Pyronaridine (PN) (a gift from Simon Croft, London School of Hygiene and Tropical Medicine, UK) and chloroquine (CQ) (Sigma-Aldrich) were dissolved in dimethylsulfoxide (DMSO). The 50% inhibitory concentrations (IC_50_) of each drug against K1 parasites were determined by the (^3^H)-hypoxanthine incorporation method [20] with minor modifications. The IC_50 _values from triplicate biological replicates (with three technical replicates in each) for PN and CQ were 11 ± 2.0 nM and 276 ± 38 nM respectively, confirming the PN-sensitive and CQ-resistant phenotypes of the K1 strain parasites. The IC_50 _values determined for the K1 strain are very similar to those reported previously [6].

For DNA microarray experiments, a synchronized culture of early trophozoite parasites (22-24 h post-invasion) was treated with the IC_50 _concentration of each drug (11 nM for PN, and 280 nM for CQ), and the final concentration of DMSO was 0.1% (v/v). For each treatment, three culture plates (90 × 15 mm) were established, each containing 1.25% haematocrit and 15-20% parasitaemia in a total volume of 17 ml. The control was a parasite culture (also synchronized and at the same stage as those used for drug treatment) containing 0.1% (v/v) DMSO, but lacking drug. Parasites were exposed to each drug for 4 and 24 h, and after each time point, parasites were harvested and liberated from erythrocytes by saponin lysis. Drug treatment was performed on 3-6 replicates. For identification of developmentally-regulated transcripts, 3 biological replicates of synchronized cultures of parasites at ring (8-10 h post-invasion), trophozoite (22-24 h post-invasion) and schizont (38-40 h post-invasion) stages were harvested for total RNA extraction.

### Total RNA extraction and cDNA labeling

Total RNA was extracted from parasites using TRIzol reagent (Invitrogen) in accordance with the manufacturer's recommendations. For each cDNA labeling reaction, 10-15 μg of total RNA were annealed with 5 μg of oligo (dT)_21 _and reverse transcribed to produce aminoallyl-dUTP(Ambion)-containing cDNA using Stratascript™ reverse transcriptase (Stratagene). The cDNA from the drug-treated RNA preparations was then labelled with monoreactive Cy5, while the cDNA from control untreated RNA samples was labelled with Cy3. To identify transcripts related to parasite development, cDNA obtained from synchronized parasites at ring, trophozoite and schizont stages were individually labelled with Cy5. A pooled reference RNA was made from the three stage samples mixed in equal proportions. The pooled reference cDNA was labelled with Cy3.

### Microarray hybridization

Purified Cy3- and Cy5-labelled cDNA were mixed in a Short Oligo Hybridization Solution (Corning Inc.), and hybridized to *P. falciparum *spotted 70-mer oligo microarrays for 16-18 h at 45°C. Arrays were washed at room temperature in a solution of 2× SSC 1× (Sodium Saline Citrate) containing 0.2% SDS, and then in 0.1× SSC. Then arrays were dried by centrifugation and immediately scanned using a ScanArray 4000B microarray scanner (Packard/Perkin Elmer, USA) or a GenePix Pro 4000 scanner according to the manufacturer's instructions. Images were analysed using ScanAlyze software [21] or Genepix Pro to obtain hybridization intensity outputs.

### Microarray data analysis

The raw intensity data from both channels were pre-processed as described previously in [22], and normalized within arrays using the global lowess algorithm and across replicate arrays from the same experiment in the Aroma package [23] run in R 2.8.1 project environment [24]. All microarray data and details of experimental design were submitted to the NCBI Gene Expression Omnibus (GEO) database and are available under series accession numbers GSE31109, GSE30867 and GSE30869.

After normalization, array features with less than three experimental values were excluded from further analyses. For study of developmentally regulated transcripts, lists were made of the average normalized log_2 _change in expression of all retained microarray features for the K1 ring, trophozoite and schizont stages (4625, 4651, and 4640 features, respectively). These lists were then compared with measurements for the corresponding microarray features from 46 time-points across the HB3 strain parasite life cycle as reported in [25] by calculation of global Pearson correlation coefficients for each K1 developmental sample and HB3 time-point.

Differentially expressed genes were identified from each drug treatment time-point using the Significance Analysis of Microarrays (SAM) programme, Excel plug-in version 3.05 [26]. Array features showing both a *q*-value of less than 5% and an expression ratio of ≥ 1.8 fold in either direction were considered as being differentially expressed (Additional file 1). The SAM algorithm uses nonparametric statistics to calculate *q*-values. The *q*-value for each feature is a measure of the positive false discovery rate (FDR), or how many features showing the same or greater level of differential expression are actually false positives [27]. Heatmap analysis of selected normalized data from array features with a significant change in expression under one or more of the drug treatment conditions was performed using the NeatMap package [28], in which rows (average normalized log_2 _change in gene expression) were clustered using the nMDS algorithm and columns (drug treatment condition) were clustered by hierarchical average linkage. Gene ontology enrichment analysis of the PN/CQ responsive genes was performed using the GOEAST web tool with default parameters [29]. Data management (filtering of gene lists) and calculation of Pearson correlation coefficients was carried out using Microsoft Excel 2007.

Statistical testing of the overlap of array features common to different drug treatments was performed using a Monte Carlo simulation method [30]. The simulation was performed to determine the null distribution of overlapping features occurring by chance among random selections of features. The simulation generated models of possible results by randomly selecting two different sets of features assumed to be the results of drug treatments. Every instance of feature overlap was tallied yielding distributions of possible outcome values. The model was constructed by randomly selecting features with replacement from the pool of 6203 independent features to reconstruct the subsets of features showing significant responses to PN (297) and CQ (205) drug treatments. The number of intersecting features between the two subsets was observed and tallied. The model is defined as thus:

Let *S *be a subset of *A *where |*A*| = *N *, the total number of features and |*S*| = *r*, the number of features in the subset *S*. *S *corresponds to a set of features chosen randomly (without replacement) from *A *using uniform distribution. Equivalently, this random process is described using the shorthand . Let *K *be the total number of simulations. The Monte Carlo simulation is described as:

for *i *= 1 to *K*:

Note that *y*(*i*) is a possible outcome of the experiment. Let *Y *be a random variable representing the intersection between *S*1 and *S*2. Let *K *be the total number of simulations. The probability of each possible intersection event can be calculated by:

In the PN/CQ Monte Carlo simulation, *K *= 1 × 10^6^; N = 6203; |*S*1| = 297; and |*S*2| = 205 respectively. The simulation was performed using a custom computer script in the Ruby interpreter programme (Additional file 2).

## Results

### Experimental design for comparative transcriptomic profiling

The goal of this study was to identify global transcriptional changes induced by PN and CQ in a CQ-resistant strain of *P. falciparum *(K1). Synchronized cultures in early trophozoite stages (22-24 h post invasion) were subjected to IC_50 _concentrations of PN (11 nM) and CQ (280 nM) for 4 and 24 h. IC_50 _drug treatments were used since previous studies of CQ transcriptional response using lower effective drug concentrations did not reveal any drug-induced transcriptional changes [31]_. _Conversely, very high drug concentrations can lead to developmental arrest which confounds analysis of the drug response (see below). Preliminary study with 30 min drug exposures did not reveal any significant changes in expression for either PN or CQ (data not shown). The 4 h time point was chosen to represent an 'early' response to the drug in which we could be reasonably confident of detecting some significant changes in expression. Importantly, the 4 h time point is also physiologically relevant with respect to pharmacokinetics, since the time taken for PN and CQ to reach their maximum plasma concentrations in patients administered with the drugs are 2-3 h for PN [32] and 2-6 h for CQ [33]. The 24 h time point was regarded as a 'late' drug response after which parasites had passed through the schizont stage, which is known to be susceptible to PN and CQ action.

To gauge the global drug-induced transcriptomic changes, DNA microarray experiments were performed in which cDNA samples from drug-treated and non-treated control cultures from each time point were labelled with different cyanine dyes, mixed and hybridized to the same microarray. In total, the dataset comprised nine microarray hybridizations each for PN and CQ.

### Comparison of PN and CQ transcriptional responses

In total, 297 and 205 microarray features were differentially expressed under PN and CQ, respectively. There is an overlap of 40 features which show significant changes in expression to both drugs (Additional file 3). To test whether this overlap is significant, a Monte Carlo simulation was performed to discover the null distribution of overlap occurring by chance for two feature lists with the corresponding number of features made randomly from the total of 6203 features that give robust signals (three or more experimental values) in all PN and CQ microarray experiments. The simulation shows that the PN and CQ overlap is highly significant (*p *≤ 1 × 10^-6^), indicating a significant similarity between the two responses. To determine the biological significance of the overlap between PN and CQ responsive features, the 254 significant features responsive to 24 h exposure of Pyrimethamine, an antifolate drug with a different mode of action [34] in the same K1 parasite strain were compared with the PN and CQ responsive features. The number of overlaps in each case was not significant (8 overlapping features for Pyrimethamine/CQ and 9 for Pyrimethamine/PN; Additional file 3).

**Figure 2:**
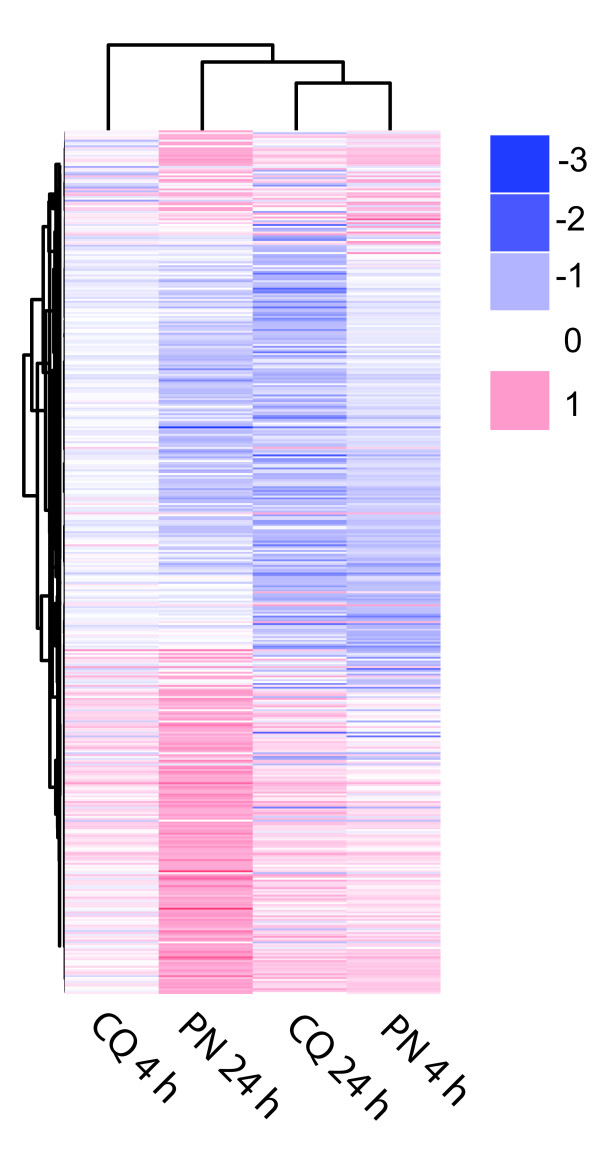
**All PN and CQ significant responsive features**. The data from 463 features showing significant changes in expression identified from microarray analysis were clustered according to the average gene expression changes as rows and the drug treatments as columns. The colour key indicates the average log_2 _fold changes in expression.

To compare the global transcriptional responses of parasites to PN and CQ after different periods of exposure, clustering and heatmap analyses were performed on the 461 features showing significant differential expression under at least one drug treatment (Figure 2). The overall transcriptional response patterns of the PN 4 h, PN 24 h and CQ 24 h treatments are similar to one another, which are confirmed by the global Pearson correlations (Table 1). The overall change in gene expression is more modest after 4 h CQ exposure compared with the others. In terms of significant differentially expressed features, far fewer were found after CQ 4 h exposure (10) than other treatments (61, 239 and 195 for PN 4 h, PN 24 h and CQ 24 h, respectively; Additional file 1).

**Table 1:** Global Pearson correlations for microarray data

	CQ 4 h	CQ 24 h	PN 4 h	PN 24 h
CQ 4 h	1			

CQ 24 h	0.62	1		

PN 4 h	0.56	0.77	1	

PN 24 h	0.62	0.73	0.65	1

Global Pearson correlation coefficients for each pairing of drug treatment conditions were calculated from the 463 features significantly responsive to PN and/or CQ.

Although many genes with significant changes in expression after drug treatment were identified, it should be appreciated that drugs typically induce cell cycle arrest, or delay before cell killing, leading to differences in cell stages between drug-treated and control cultures. During normal growth, parasite stages show markedly different gene expression profiles from one another [25,35], and so the gene expression profile of drug-treated parasites will likely differ from control untreated parasites in part because of drug-induced developmental delay. Different anti-malarial drugs induce cell cycle arrest to different extents; for example, lethal concentrations of antifolates do not induce morphological arrest [36]. On the other hand, the developmental arrest induced by cytostatic drugs such as DL-α-difluoromethylornithine necessitate complex experimental designs employing multiple time-points of drug-treated and control cultures in order to correct for transcriptional changes relating only to differences in cell stage [37].

**Figure 3:**
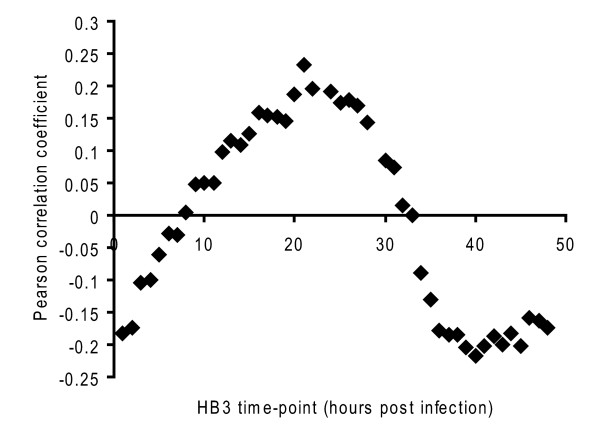
**Correlation between K1 Trophozoite (22-24 h) and intraerythrocytic developmental cycle data for strain HB3**.

To identify the developmentally regulated genes, which could confound identification of PN and CQ-responsive genes, RNA samples from drug-free synchronized cultures from ring, trophozoite, and schizont stages were individually labelled and hybridized with a pooled sample from the three stages. The data from this experiment were used to compare the developmental profile of the K1 strain with the high-resolution data for the HB3 strain (Additional file 4). The peak of Pearson correlation between the K1 trophozoite culture sample (estimated age 22-24 h post infection) and HB3 is maximal at HB3 time-points 20-24 h (Figure 3), indicating a high concordance between the two strains in terms of developmentally regulated transcripts.

After 4 h drug treatment, > 90% of parasites were at the trophozoite stage, while after 24 h drug treatment parasites were at schizont and early ring stages. Therefore, microarray features showing developmental change in the HB3 dataset (1.8 fold or greater change in expression) were filtered out from the lists of features differentially expressed under drug treatment. For the 4 h drug treatments, features which were found to be HB3 developmentally regulated at any of the time-points 22-28 h post infection were removed. For the 24 h drug treatments, features developmentally regulated at any of the HB3 time-points 22-48 h and 1-4 h post infection were removed. This filtering, therefore, establishes a subset of differentially expressed features in which features showing expression changes solely due to differences in staging during the course of drug exposure have been removed. In total, there were 145 features after developmental filtering which showed significantly different expression under at least one of the drug exposure conditions tested (Additional file 5).

**Figure 4:**
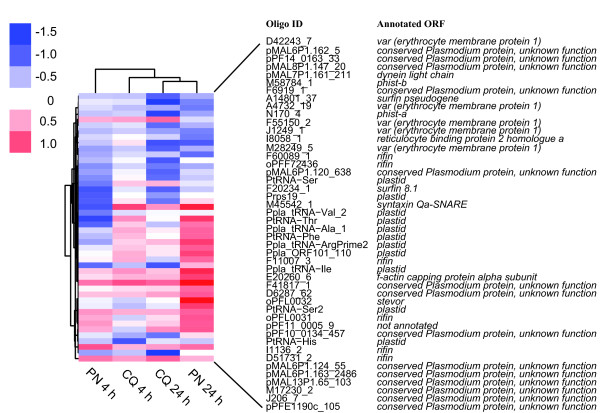
**PN and CQ responsive features after filtering of developmental and secondary responsive features**. The data from 46 features which show significant changes in expression under CQ and/or PN exposure after filtering identified from microarray were clustered according to the average gene expression changes as rows and the drug treatments as columns. The colour key indicates the log_2 _fold changes in expression. The feature oligo IDs and gene annotations (Plasmodb v8.0) are shown on the right.

Developmental arrest induced by drug action is but one confounding factor in the analysis of drug-induced transcriptional profiles. In order to gain insight into drug mechanism of action from microarray data, the primary or direct effects of the drug need to separated from indirect (e.g. general stress response), secondary and bystander effects (reviewed in [38]). A recent *P. falciparum *microarray study identified a set of 3705 "perturbation" responsive genes which change expression in response to widely different drugs and other stresses [39]. The perturbation gene set was then used to filter out features likely responding to drug in an indirect, or secondary fashion. 72 PN/CQ responsive features with gene annotations matching the perturbation gene list were removed by this filtering step. Another indirect genetic response to drug action is activation of gametocyte stage genes [40]. 26 PN/CQ features with gene annotations matching the list of genes up-regulated (p-Anova < 0.05) in gametocyte stages [41] were removed by this final filtering step, leaving 46 primary PN/CQ responsive features. The heatmap analysis of these features in Figure 4 shows overall similarity of PN and CQ responses. Inspection of the annotated genes for these features reveals a striking abundance of genes encoding host-exported proteins of different families, i.e. *var, stevor, surfin, rifin *and *phist*, which is supported by the significant gene ontology terms (Table 2).

**Table 2:** Enriched Gene ontology terms represented by the 46 PN/CQ features after filtering

GO ID	Ontology	Term	q	m	T	K	genes	log_odds_ratio	p
GO:0005576	cellular_component	extracellular region	7	221	2198	10	PFA0765c//MAL7P1.57//PFE0065w//PFI0005w//PFF0855c//PFL2640c//PFL2620w	2.7995	0.000272

GO:0018995	cellular_component	host	7	220	2198	10	PFA0765c//MAL7P1.57//PFE0065w//PFI0005w//PFF0855c//PFL2640c//PFL2620w	2.806043	0.000272

GO:0020002	cellular_component	host cell plasma membrane	5	190	2198	10	PFA0765c//MAL7P1.57//PFI0005w//PFF0855c//PFL2640c	2.53212	0.01505

GO:0033643	cellular_component	host cell part	7	220	2198	10	PFA0765c//MAL7P1.57//PFE0065w//PFI0005w//PFF0855c//PFL2640c//PFL2620w	2.806043	0.000272

Significantly enriched gene ontology terms were identified using the GOEAST tool. The top four significant terms are shown. GO ID is the identifier for the GO term in the Gene Ontology Project, Ontology represents the ontology category the GO term belongs to, term is the GO definition, q is the number of genes associated with the same GO ID in the data analyzed, m is the total number of genes associated with the listed GO ID in the genome, t is the total number of annotated genes for *Plasmodium falciparum*, k is the total number of features with gene annotations considered for analysis, genes are the Gene IDs sharing the same significant GO ID, log_odds_ratio is the log_2 _odds ratio of the GO ID enrichment and p is the FDR-adjusted *p*-value for significance.

## Discussion

Although PN is well-recognized as a highly potent anti-malarial drug against both sensitive and multi-drug resistant lines of malaria parasites, its molecular mechanism of action is not fully understood. Previous studies have demonstrated that its primary mode of action is the same as CQ, namely interaction with haem and subsequent inhibition of β-haematin assembly. Given that both drugs are structurally related and are proposed to act on the same target(s), it was hypothesized that they may induce similar transcriptomic changes at IC_50 _concentrations.

The transcriptome of chloroquine-resistant *P. falciparum *K1 strain in response to PN and CQ exposure was investigated. Similar to previous microarray studies of *P. falciparum *under anti-malarial drug treatment [36,42], the magnitude of change in expression for most genes in response to CQ and PN was low. Nonetheless, 297 features showed significant change to PN and 205 to CQ. The overlap of 40 features among the PN and CQ features was highly significant, suggestive of a similar genetic response. In contrast, the PN and CQ features overlapping with those responsive to an unrelated drug, Pyrimethamine in the same K1 parasite strain exposed for 24 hours were not significant. In total, 461 features showed significant changes in response to either or both PN and CQ drugs. A major difficulty in interpreting these data with respect to primary mechanism of drug action is confounding responses not directly related to the mechanism of drug action. Although no delay of parasite development was observed in CQ and PN drug-treated cultures by comparison of morphology, it is known that transcriptional arrest precedes morphological arrest [37]. It should be noted that the term transcriptional arrest relates to a delay in the stage-specific pattern of gene expression within a short time-frame, i.e. the same 48 h developmental cycle. This phenomenon is probably quite distinct from the changes occurring in recrudescing parasites in the longer time-scale of a clinical treatment period (3-4 days). Such a delay in the developmental programme of gene expression may, therefore, have a confounding effect on identification of drug-specific responders. A conservative approach was taken to address this confounding effect, in which all stage-specific transcripts from the corresponding developmental stages during the different drug treatment periods were removed. This approach, however, could lead to removal of genes which are primary drug responsive, yet developmentally regulated.

Besides the primary effects of PN and CQ treatment, i.e. inhibition of α-haematin formation and glutathione-dependent haem degradation, secondary/downstream effects of these drugs on the parasite have been described. At very high concentrations, CQ and other quinoline drugs disrupt vesicular transport [43,44]. Moreover, CQ inhibits translation [45], disrupts mitochondrial membrane potential and induces DNA fragmentation [46]. The PN and CQ responsive microarray features with gene annotations matching the perturbation dataset [39] could be considered secondary responses. The gene annotations encompass a variety of cellular functions, although none are significantly associated with the aforementioned CQ secondary effects. Microarray features corresponding to the *Pfcrt *gene, which mediates resistance to CQ, showed significant changes in expression in response to PN and CQ. However, the *Pfcrt *gene is present in the perturbation dataset and cannot be considered as a primary PN or CQ responsive gene. Moreover, no significant changes in expression were found for features corresponding to other CQ and quinoline drug transporter genes reported in the literature (*Pfmdr1, Pfmdr 2, Pfmrp1, Pfmrp2 *and *Pfnhe1*).

After filtering by comparison with the perturbation dataset and removal of genes known to be up-regulated in gametogenesis, the remaining 46 PN and CQ responsive features showed a significant over-representation of genes encoding host-exported proteins. These genes belong to large gene families, including *var, stevor, surfin, rifin *and *phist*. A previous study of the transcriptional response of *P. falciparum *under IC_50 _CQ treatment showed that 36 genes could be considered as primary CQ responsive, with many more genes showing changes in expression because of differences in staging and secondary effects [47]. Of the 36 primary CQ genes reported in [47], two *rifin *genes (PF10_0006 and PF10_0404) are also present among the annotated genes represented by the 46 PN/CQ responsive features after filtering. Although the data are suggestive of primary PN and CQ responsive genes, other perturbations such as antifolates [36], and the bisthiazolium compound T4 [40] also induce changes in expression of host-exported proteins of multi-gene families, albeit for separate genes. Despite some common features of transcriptional regulation, including epigenetic switching mechanisms and sharing of transcriptional activators [48], very little is known of how the expressions of these gene families are modulated by environmental perturbation. Given that different drugs exert responses in these gene families, their roles as potential mediators of drug resistance should be investigated further.

Comparison of the drug-responsive genes shows that PN appears to exert a faster-acting transcriptional perturbation than CQ. From the four drug treatment conditions tested in this study, the PN 4 h treatment was globally more similar to the CQ 24 h treatment than CQ 4 h. Similarities between the PN and CQ transcriptional responses were also noted in overlapping genes and functional categories, which support the hypothesis that the genes responsive to the two drugs are similar. It should be noted that because PN is far more potent than CQ against the K1 strain, much higher concentrations of the latter drug were needed to produce similar biological effects. Therefore, the differences in cytosolic concentrations between PN and CQ may contribute to some of the minor observed differences between the PN and CQ transcriptional responses. A simple explanation for why PN is faster acting and thus more effective than CQ, especially against CQ-resistant parasites, can be made based on the known chemical properties. PN is more hydrophobic than CQ, with a log P value comparable to amodiaquine [49,50] and thus the uptake into the food vacuole, the site of action, will be faster for PN. Furthermore, PN has an extra highly basic group, which means it may be protonated more readily and thus accumulate more rapidly in the food vacuole than CQ.

## Conclusions

This study is the first to report global perturbations on *P. falciparum *gene expression in response to IC_50 _concentration of PN. Since the primary modes of action for PN and CQ are thought to be the same, expression changes induced by CQ were studied for comparison. The genetic response to PN and CQ are similar, although PN appears to exert a faster and more pronounced effect than CQ since the transcriptomic changes are discernable after a 4 h exposure of PN, whereas a longer exposure is needed for a comparable effect with CQ. It is not possible, however, to relate the drug-responsive genes with the mode of action of these drugs, since the majority of genes changing expression can be related with drug-induced developmental arrest and general response to perturbation.

## Competing interests

The authors declare that they have no competing interests.

## Authors' contributions

KK performed drug-response microarray experiments, data processing and analysis. CW performed cell-cycle microarray experiments and processed data. SC and PJS analysed data, prepared figures and wrote the manuscript. PCP and SK analysed data and helped drafting and editing of the manuscript. All authors read and approved the manuscript.

## Supplementary Material

Additional file 1 **PN and CQ significant responsive features**. Microarray features showing significant changes in expression called by the Statistical Analysis of Microarrays algorithm under each PN and CQ treatment condition. Click here for file

Additional file 2 **Null distribution of overlapping microarray features from PN and CQ drug treatments determined by Monte Carlo modelling**. Click here for file

Additional file 3 **Comparison of features showing significant changes in expression under different drug treatments**. Click here for file

Additional file 4 **Comparison of transcriptional changes between K1 and HB3 intraerythrocytic stages**. Click here for file

Additional file 5 **Filtering of PN and CQ features (i) developmentally regulated across treatment periods identified by matching to HB3 dataset (ii) perturbation responsive identified from Hu et al. dataset (iii) upregulated in gametocyte stages, identified from Young et al. dataset**. See Materials and methods for details. Click here for file
